# Feeling Stressed or Strained? A Biophysical Model for Cell Wall Mechanosensing in Plants

**DOI:** 10.3389/fpls.2019.00757

**Published:** 2019-06-12

**Authors:** Antoine Fruleux, Stéphane Verger, Arezki Boudaoud

**Affiliations:** ^1^Laboratoire de Reproduction et Développement des Plantes, Université de Lyon, ENS de Lyon, UCBL, INRA, CNRS, Lyon, France; ^2^Department of Forest Genetics and Plant Physiology, Umeå Plant Science Centre, Swedish University of Agricultural Sciences, Umeå, Sweden

**Keywords:** mechanosensing, mechanical signal, stress, strain, cell wall, biophysical model, plants

## Abstract

Mechanical signals have recently emerged as a major cue in plant morphogenesis, notably influencing cytoskeleton organization, gene expression, protein polarity, or cell division. Although many putative mechanosensing proteins have been identified, it is unclear what mechanical cue they might sense and how this would occur. Here we briefly explain the notions of mechanical stress and strain. We present the challenges to understand their sensing by plants, focusing on the cell wall and the plasma membrane, and we review putative mechanosensing structures. We propose minimal biophysical models of mechanosensing, revealing the modes of mechanosensing according to mechanosensor lifetime, threshold force for mechanosensor dissociation, and type of association between the mechanosensor and the cell wall, as the sensor may be associated to a major load-bearing structure such as cellulose or to a minor load-bearing structure such as pectins or the plasma membrane. Permanent strain, permanent expansion, and relatively slow variations thereof are sensed in all cases; variations of stress are sensed in all cases; permanent stress is sensed only in the following specific cases: sensors associated to minor load-bearing structures slowly relaxing in a growing wall, long-lived sensors with high dissociation force and associated to major-load-bearing structures, and sensors with low dissociation force associated to major-load-baring structures behaving elastically. We also find that all sensors respond to variations in the composition or the mechanical properties of the cell wall. The level of sensing is modulated by the properties of all of mechanosensor, cell wall components, and plasma membrane. Although our models are minimal and not fully realistic, our results yield a framework to start investigating the possible functions of putative mechanosensors.

## Introduction

**FIGURE 1:**
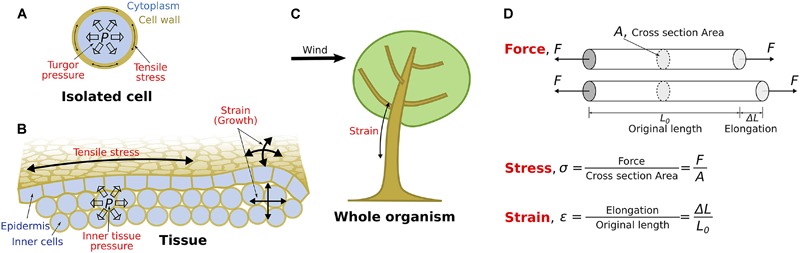
Mechanical signals and physical quantities. **(A–C)** Schematic representation of common occurrences of stress and strain as sources of mechanical signals at the cell **(A)**, tissue **(B)**, and whole organism level **(C)**. **(A)** At the single cell level, turgor pressure from the cytoplasm leads to tensile stress in the cell wall. **(B)** At the tissue level (here epidermis and two inner cell layers), inner tissue pressure may lead to tensile stress and growth in the epidermis. **(C)** At the whole organism level (here a tree exposed to wind), external forces can induce significant strain. **(D)** Schematic and mathematical representation of the notions of force, stress and strain. Forces *(F)* are represented as vectors applied in opposite directions at both ends of the object. Stress (σ) is the ratio of the force *(F)* to the cross-sectional area *(A)* of the object on which the force is applied. Strain (𝜀) is the ratio of the elongation of the object (Δ*L*) to its original length *(L_0_)*.

All living organisms experience mechanical stress, associated with internal or external mechanical forces, and strain (deformation) resulting from such forces. For instance, in plants, the turgor pressure generated inside individual cells puts the cell wall under tension (Figure 1A). This tensional stress is the driving force for growth (Figure 1B), but if it is not properly managed, it can lead to cell bursting (Sapala et al., 2018). In developing aerial tissues, tension builds up in the epidermis (Figure 1B; Peters and Tomos, 1996; Kutschera and Niklas, 2007; Beauzamy et al., 2015; Verger et al., 2018). This tissue stress can affect the integrity of the organism, notably by pulling its cells apart (Galletti et al., 2016; Verger et al., 2018). Tissue stress patterns may also be more complex. For example, in developing wood, the vascular cambium (secondary meristem responsible for wood formation) is both under axial directional tensile stress (Hejnowicz, 1980) and is under compression between a rigid lignified layer of bark and the lignified wood (Fischer et al., 2019). External forces, which can induce significant stress and strain (Figure 1C), are also a threat to the organism since they may damage the tissues (Moulia et al., 2015). For instance, strong winds induce stem breakage (Eloy, 2011; Moore et al., 2018). Accordingly, perception of mechanical forces may have emerged from the need to maintain the physical integrity of cells and organisms (Moulia et al., 2015), which would in turn influence growth and morphogenesis (Mirabet et al., 2011; Zhao et al., 2018). There are numerous examples of plant responses to mechanical forces, from molecular and cellular level – reorganization of the cytoskeleton (Landrein and Hamant, 2013; Robinson and Kuhlemeier, 2018), calcium influx (Trewavas and Knight, 1994; Monshausen and Haswell, 2013), and changes in protein polarity or in gene expression (Lee et al., 2005; Bringmann and Bergmann, 2017) – to tissue and organism levels – organogenesis (Ditengou et al., 2008; Hamant et al., 2008; Heisler et al., 2010; Nakayama et al., 2012; Landrein et al., 2015), fast movements (Poppinga et al., 2016), formation of tension and compression wood (Okuyama et al., 1994) and control of plant posture (Eloy, 2011; Moore et al., 2018).

Despite continuous progress in understanding the molecular basis of mechanosensation and its consequences on macroscopic phenotypes, there is an ongoing debate concerning how and whether plants sense stress and/or strain (see replies to Hamant et al., 2008 by Schopfer and Meyerowitz in science e-letters; Heisler et al., 2010; Nakayama et al., 2012; Bozorg et al., 2014; Moulia et al., 2015). More generally, it is still unknown what cue or combination of cues is sensed and how does a putative mechanosensing protein or molecule react to such cue.

Here, we focus on mechanosensing at the plant cell wall and aim at providing hypotheses about how mechanosensors associated with the cell wall might work. Following an introduction to mechanical signals, we review putative mechanosensors based on the literature. We then propose parsimonious biophysical models to investigate how such mechanosensors could be involved in sensing stress, strain, and/or a combination of both. These models provide a framework to examine the links between cell wall level stress and strain (“macroscopic” scale) and changes in state/binding/unbinding/deformations of mechanosensors (“microscopic” scale). We finally discuss how this framework might shed light on the role of the different classes of putative mechanosensors.

## Mechanical Signals

Mechanical signals, such as stress and strain, are physical quantities that are difficult to fully comprehend, and so are often confused with each other. The notion of force is intuitive: to exert a force on an object is, in absence of compensation by an opposite force, to give motion to this object. Force quantifies the propensity to give motion. A force is represented by a vector (Figure 1D) and expressed in newtons (N). Two compensating forces quantify the propensity of an object to deform, i.e., change in dimensions or lengths (Figure 1D). For instance, a rope is deformed when pulled from its two ends by forces of opposite directions and same magnitude. Actually, the deformation of the rope does not only depend on the magnitude of the force but rather depends on the ratio of the force to the cross-sectional area of the rope – this ratio is known as mechanical stress (Figure 1D). Indeed, the rope resists more the applied force if it is thicker, because each of its fibers bears less force when the rope is thicker. Accordingly, applying the same force to two ropes, equivalent in nature, but of different diameter, will result in higher stress in the thinner rope than in the thicker one. Stress is the local equivalent of the force. Stress is not represented by a vector, but by a mathematical object known as a tensor, because it involves the direction of force and the orientation of the cross-section to which the force is applied (Boudaoud, 2010). It is expressed in newton per square meter (N/m^2^) or pascal (Pa). As exemplified above, stress is the appropriate quantity when considering the deformation of an object.

Strain is the measurable deformation of an object that can arise due to applied stress (Figure 1D). Strain can be quantified as the relative change in dimensions of the object, and so has no physical units. Strain is also represented by a tensor. Note, however, that it will be hereafter sufficient to consider configurations in which stress and strain are unidirectional. In such configuration, strain can be expressed as (*L*-*L*_0_)/*L*_0_ = Δ*L/L_0_* (Figure 1D), where *L*_0_ and *L* are the lengths of the object at rest and deformed, respectively, and Δ*L = L-L_0_* is the elongation of the object.

The material of which the object is made can have different properties concerning stress and strain. The material is elastic if the object returns to its initial state after applied stress is released. An elastic material is linearly elastic if stress is a linear function of strain, a relation known as Hooke’s Law; a spring is typically made of a linearly elastic material. The Young’s modulus, which is expressed in pascals, is the main parameter of the Hooke’s law; a material is soft or stiff if it has a small or a large Young’s modulus. If stress depends on the rate at which strain is increased, then the material is considered as viscoelastic, else as purely elastic. In addition, most materials lose elastic behavior when stress is increased above a threshold; deformations become irreversible – some of the deformation remains after stress has been released – and the material is considered as plastic if it has not ruptured. If stress depends on the rate of irreversible deformations then the material is considered as viscoplastic. The plant cell wall is effectively a viscoelastoplastic material: it behaves as a viscoelastic material when stress is below the yield threshold and as a viscoplastic material above this threshold (Boudaoud, 2010; Cosgrove, 2018a), though the mechanics of the cell wall is coupled to its chemistry (synthesis and remodeling). In line with previous models of cell wall expansion (Lockhart, 1965; Green et al., 1971; Ortega, 1985; Proseus et al., 1999; Munns et al., 2000), we use a viscoelastoplastic description in the biophysical model that we introduce below.

In physical systems, strain is directly quantified from changes in object dimensions, while stress is often measured using the deformation of a body of known shape and material, such as in some pressure gauges and manometers, and material properties (Young’s modulus for an elastic material, viscosity for a viscous material, etc.) are deduced from the quantification of stress and strain. In living systems, the simplest hypothesis would be that the deformation of mechanosensitive molecules would induce downstream events and therefore that mechanosensors would measure strain. However, living systems are highly heterogeneous and mechanosensitive molecules are very small. It is thus unclear how strain measured at molecule level is related to mechanical signals at supramolecular scale, and whether molecule deformation mainly depends on strain, on stress, or on another mechanical quantity defined at a higher scale, such as cell or tissue scale.

In plants, the cell wall is the stiffest component of cells and tissues; it is the main stress-bearing component and is crucial for the mechanical integrity of the plant. Accordingly, perception of mechanical forces is expected to primarily take place at the cell wall and associated structures.

## Putative Mechanosensors

Mechanosensing has received considerable attention, leading to the discovery of many mechanosensing mechanisms (Orr et al., 2006; Kumamoto, 2008; Rodicio and Heinisch, 2010; Hamant and Haswell, 2017). The general mechanism consists in the mechanically induced deformation of a sensor (e.g., unfolding, opening of a channel, change of chemical affinity, etc.) causing a chemical signal (e.g., unmasking of a binding site, ion influx, release of a signaling molecule, etc.), effectively transforming a mechanical information into a chemical information that can be integrated by the cell in order to respond to this signal. Interestingly, sensing by a single molecule occurs at microscopic scale and reflects the mechanical signal in the close neighborhood of the molecule, whereas the downstream chemical signal can be, in principle, averaged from multiple sensors at local (subcellular) level or at more global level (cell, tissue) (Bouffanais et al., 2013; Étienne et al., 2015).

Mechanosensors come in various forms. In order to better understand how plants could sense mechanical signals, we review hereafter some of the structures and mechanisms that are associated with the cell wall and that have been implicated or are suspected to be implicated in cell wall mechanosensing.

### The Cell Wall

**FIGURE 2:**
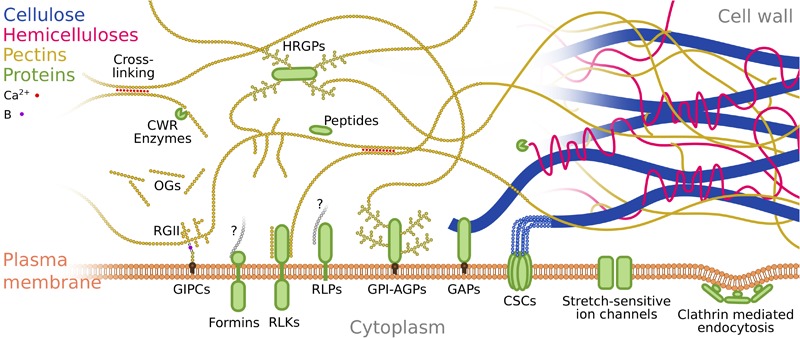
Putative mechanosensing structures at the cell wall and plasma membrane. Schematic representation of the various structures potentially involved in plant mechanosensing, and their interactions. For clarity and ease of representation we focus on some of the interactions between pectins, proteins and plasma membrane (and focus less on cellulose and hemicelluloses). Cellulose is represented in blue, hemicelluloses in magenta, pectins in yellow, proteins in green and plasma membrane in orange. Red dots represent calcium ions involved in pectin cross linking. The purple dot represents boron involved in RGII-GIPC cross-linking. Gray polysaccharides labeled with a question mark in the case of Formins and RLPs accounts for interaction of these proteins with the cell wall with the nature of the polysaccharide or molecule involved remaining unknown. Ca^2+^, Calcium; B, Boron; CWR, Cell Wall Remodeling; HRGP, Hydroxyproline-rich Glycoproteins; OGs, OligoGalacturonans; RGII, RhamnoGalacturonanII; GIPCs, Glycosyl Inositol Phospho Ceramides; RLKs, Receptor-Like Kinases; RLPs, Receptor-Like Proteins; GPI-AGPs, GlycosylPhosphatidylInositol anchored ArabinoGalactan Proteins; GAPs, GlycosylPhosphatidylInositol Anchored Proteins; CSCs, Cellulose Synthase Complexes.

The cell wall of developing plants cells is a very dynamic compartment which undergoes constant synthesis and remodeling, and which hosts a large diversity of proteins. It is mainly made of cellulose, hemicelluloses and pectins (Figure 2; Burton et al., 2010). These are polymeric chains of sugars (polysaccharides) that have various sugar composition and structures. Most polysaccharides can interact or bind with each other as well as with cell wall proteins (Tan et al., 2013; Höfte and Voxeur, 2017), forming a viscoelastoplastic network of polysaccharides and proteins. Mechanical signals could have an effect on any of its constituents. For example, stress or strain could induce cleavage of polysaccharides, potentially leading to the formation of oligosaccharides (fragments of polysaccharides) that can then be sensed by membrane receptors to trigger a chemical signal (Kohorn et al., 2009; Brutus et al., 2010). In addition tension or stretching of these polysaccharides may change their chemical properties and affinities with other components of the cell wall (Boyer, 2009). The cell wall contains numerous and diverse proteins (Jamet et al., 2008). For instance, Hydroxyproline-Rich cell wall Glycoproteins (HRGPs) are covalently linked with other cell wall components through their glycans (Figure 2; Tan et al., 2013; Showalter and Basu, 2016). Such proteins contribute to bearing cell wall stress and might be involved in sensing by mechanisms such as unfolding or change in chemical affinity of their protein domain. Cell wall localized peptides, such as the Rapid Alkalinization Factors (RALFs) can be trapped by non-covalent bonds to cell wall components (Murphy and De Smet, 2014; Mecchia et al., 2017). Stress or strain of these components might reduce their affinity to peptides, leading to peptide release and subsequent perception by membrane receptors (Haruta et al., 2014). Finally, the affinity of cell wall remodeling and hydrolyzing enzymes (e.g., pectin methylesterases, polygalacturonases, xyloglucan endotransglucosylase/hydrolase, expansins, peroxidases, etc.) to their substrate might depend on stress or strain of the substrate. The consequent change of enzyme activity could also lead to release of oligosaccharides that would then be sensed by membrane receptors. This non-comprehensive list of mechanisms reveals the mechanosensing potential for the cell wall and its components (Figure 2). Nevertheless, there is no clear evidence for such mechanism so far.

The diversity of potentially mechanosensitive structures in the plant cell wall also reflects a diversity of mechanical properties. For instance the cellulose microfibrils, made of 18 linear chains of ß-1,4 linked glucose, are extremely stiff (Cosgrove, 2018b). Other polysaccharides such as hemicelluloses and pectins are made of single linear chains with lateral branches, and are individually much less stiff than cellulose microfibrils. However, the cross-linking capacity of most polysaccharides allows a range of large-scale mechanical properties as they assemble into more or less densely cross-linked networks (Willats et al., 2001). Finally, structural proteins are diverse in structure and might show a range of deformability. Assuming that most or all of these components are directly or indirectly involved in mechanosensing, this diversity of mechanical properties could yield different types of mechanosensing.

### The Cell Wall-Plasma Membrane Continuum

Arguably, the most studied type of mechanosensors are the plasma membrane stretch-sensitive ion channels. They are widespread across living kingdoms (Peyronnet et al., 2014; Basu and Haswell, 2017). These channels are protein complexes inserted in the plasma membrane and arranged in a structure forming a pore. This pore is closed when the membrane is under low tension. Conversely, high enough tension in the membrane leads to rearrangement of the protein complex and opening of the pore. This allows entry of ions such as calcium in the cell, and changes in ion intracellular concentration can then be sensed. Plants possess various types of plasma membrane stretch-sensitive ion channels (Figure 2) including the McS-Like (MSL), Mid1-Complementing Activity (MCA), Two Pore Potassium (TPK), Reduced hyperosmolarity-induced [Ca^2+^] increase (OSCA), Piezo and possibly Defective Kernel1 (DEK1) (Hamant and Haswell, 2017; Guerringue et al., 2018). Experimental work has shown that some of them are indeed involved in mechanosensing. This may seem surprising considering that the cell wall is much stiffer than the membrane, so that the membrane bears very little tension compared to the cell wall.

A possible explanation relies on the numerous direct and indirect links between the cell wall and the plasma membrane, that would propagate cell wall stress or strain to the plasma membrane tension (Figure 2; Liu et al., 2015). Indeed, the plasma membrane contains a number of transmembrane Receptor-Like Kinases (RLKs) and Receptor-Like Proteins (RLPs), such as the Wall-Associated Kinases (WAKs), *Catharanthus roseus* RLK1-Like Kinases (CrRLKs), Proline-rich Extensin-Like Receptor Kinases (PERKs), Lectin receptor-like kinases and Formins that have been shown to interact with the cell wall (Wolf, 2017). In addition to providing a link between cell wall and plasma membrane, these proteins could also act as mechanosensors (Shih et al., 2014; Engelsdorf et al., 2018). Among these proteins, the WAKs and the CrRLKs have been shown to interact with the homogalacturonan fraction of the pectin specifically (Decreux and Messiaen, 2005; Feng et al., 2018). Another interesting family of proteins are the cellulose synthases, which form a transmembrane protein complex synthesizing cellulose while at the plasma membrane, intrinsically creating a link between cellulose in the cell wall and the plasma membrane (Somerville, 2006). In addition to transmembrane proteins, Glycosylphosphatidylinositol (GPI) anchored proteins (GAPs) can provide a link between the cell wall and plasma membrane. The GPI is a glycolipid post-translational modification that allows the anchorage of the protein to the plasma membrane. GPI-ArabinoGalactan Proteins (GPI-AGPs) are a subfamily of HRGPs mentioned above and thus may bind to cell wall polysaccharides (Showalter and Basu, 2016). Another family of GAPs is the COBRA family which has the capacity to bind to crystallized cellulose *in vitro* (Sorek et al., 2014). Finally the plasma membrane can be directly linked to the cell wall by glycosylated lipids called Glycosyl Inositol Phospho Ceramides (GIPC); their extracellular glycans interact with RhamnoGalacturonanII, a type of pectins, in the presence of boron (Voxeur and Fry, 2014).

In addition to stretch-activated channels, mechanosensing at the plasma-membrane could involve other mechanisms. For instance endocytosis and exocytosis are affected by the level of membrane tension (Gauthier et al., 2012). Endocytosis requires the invagination of the membrane inside the cell in order to form a vesicle. Endocytosis is mostly mediated by the binding, through adaptor proteins, of clathrins to ligands on the intracellular membrane surface, which bends the membrane due to the shape of clathrin complexes. But this process can in principle be counteracted by membrane tension which maintains the membrane as flat as possible. Thus clathrin could directly act as membrane tension sensor as their capacity to bend the membrane would decrease with increasing membrane tension.

### Processes That Modulate Mechanosensation

A mechanosensor, whether it is a protein, a protein complex or a polysaccharide for example, needs to be part of, inserted in, or linked with a medium in order to sense mechanical signals within this medium. This medium could be the cell wall, subparts of the cell wall (cellulose, hemicelluloses, pectins, etc.), or the plasma membrane. How the mechanosensor is coupled to the medium will impact on the extent of force transferred from the medium to the sensor. Another important process is the turnover of the sensor. The sensor can be dynamically inserted in and removed from the medium, undergo dissociation, or have a reversible activation; kinetic parameters of these processes will influence mechanosensation. Finally the response to the mechanical signal is likely not triggered by a single sensor, but rather by the averaging and filtering of the signal created at the microscopic scale by a large number of sensors. Coupling, turnover, and integration yield a complex network of processes from which mechanosensing emerges. As a consequence, a biophysical model may give more insight into how mechanosensing actually works, what type of signal (stress, strain, and/or a combination) can be sensed, and what may be the role of each type of mechanosensor.

## A Biophysical Model for the Cell Wall and for Mechanosensing

**FIGURE 3:**
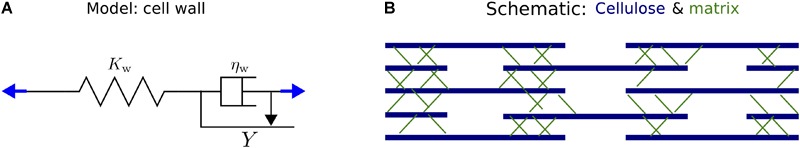
A mechanical model for the cell wall. **(A)** The cell wall is mechanically represented as a spring of elastic modulus K_w_ and a dashpot of viscosity η_w_ in series. **(B)** The corresponding schematic of cell wall structure with cellulose fibers and the matrix shown in blue and green, respectively.

**FIGURE 4:**
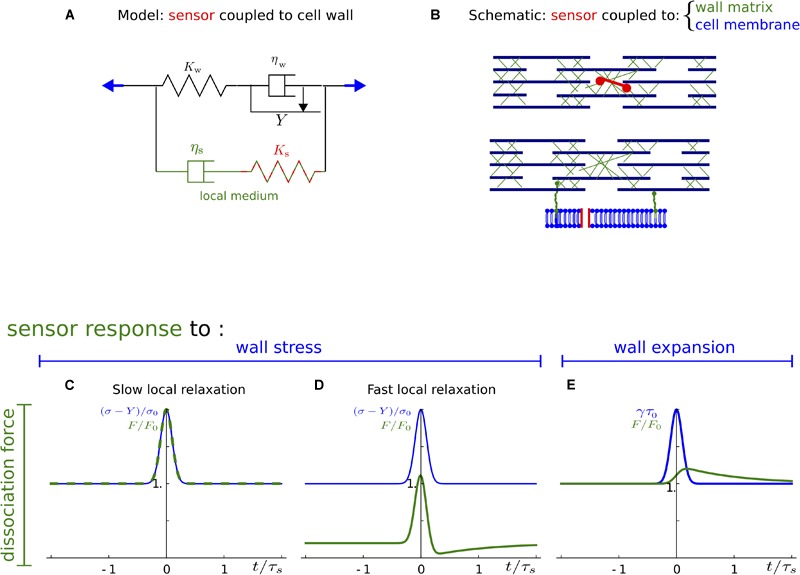
Sensor associated with a minor load-bearing structure. **(A)** The sensor and the structure with which it is associated are schematically represented by a spring in parallel to the cell wall. The elastic modulus K_s_ accounts for the sensor and the structure in which it is inserted. The viscosity η_s_ is a property of the structure, only. **(B)** Schematic of the cell wall showing that the minor load-bearing structure may be a matrix component or the plasma membrane. **(C–E)** Response of the sensor to a mechanical signal: stress **(C,D)** or strain/expansion **(E)**. Mechanical signal (input, blue) and dissociation force (output, green) are plotted as a function of time. Stress is normalized using the yield threshold, *Y*; and an arbitrary stress level, σ_0_; expansion rate is normalized by 1/τ_0_ = σ_0_/(*K_w_τ_s_*); dissociation force is normalized by F_0_ = K_s_/K_w_σ_0_; time is normalized with local relaxation time τ_s_. **(C,D)** Correspond to the ratios τ_s_/τ_w_ = 2 and τ_s_/τ_w_ = 1 of local to cell wall relaxation time, respectively.

**FIGURE 5:**
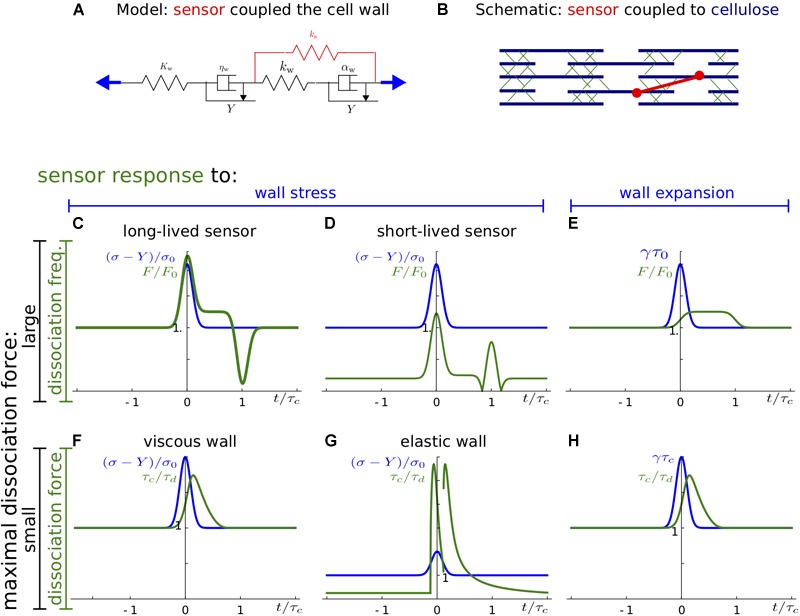
Sensor associated with a major load-bearing structure. **(A)** The sensor is schematically represented as a spring in parallel to a small segment of the cell wall. K_s_ is the spring constant of the sensor and K_w_ and η_w_ are the spring constant and the viscous coefficient of the corresponding segment of cell wall. **(B)** Schematic of the cell wall showing that the major load-bearing structure may be cellulose microfibrils. **(C–H)** Response of the sensor to a mechanical signal: stress **(C,D,F–G)** or strain/expansion **(E,H)**, with large **(C–E)**, and small **(F–H)** dissociation force. Mechanical signal (input, blue) and dissociation force (output, green) are plotted as a function of time. Stress is normalized using the yield threshold, *Y*; and an arbitrary stress level, σ_0_; expansion rate is normalized by 1/τ_0_ = σ_0_/(*K_w_τ_c_*) in **(E)** and by the characteristic dissociation rate 1/τ_c_ in **(H)**; dissociation force is normalized by F_0_ = K_s_/K_w_σ_0_; dissociation frequency 1/τ_d_ is normalized by 1/τ_c_; time is normalized by the dissociation time τ_c_. **(C,D)** Correspond to the ratios τ_s_/τ_w_ = 0.2 and τ_s_/τ_w_ = 1 of local to cell wall relaxation time, respectively. **(F,G)** correspond to a viscous and an elastic behavior of the wall. The value of dissociation force is equal to 0.5k_s_/k_w_σ_0_, 0.5τ_c_/η_w_σ_0_, and 0.5k_c_ in **(F–H)**, respectively.

We propose a simplified one-dimensional description in which the mechanical properties of a sensor, of the plasma membrane, and of the cell wall are symbolically represented by springs, dashpots, and frictional blocks (Figure 3A, 4A, 5A), which stand for the elastic, viscous, and plastic behavior of the sensor or of the structure, respectively. In the following, we introduce the models for the cell wall and the sensors, and then investigate the response of the sensors to loading. The details of the model and calculations are given in the Supplementary Material.

### Model Ingredients and Assumptions

#### The Cell Wall

The Lockhart model (Lockhart, 1965) relies on a commonly accepted description of the cell wall as a viscoelastoplastic material. We adopt this description of the cell wall, which in 1D can be represented as the association of a spring, a dashpot, and a frictional block (Figure 3A,B). When the tension in the wall is below the yield stress, Y, the wall behaves as a spring of stiffness K_w_; when tension is above the threshold, Y, the wall behaves like a dashpot of viscosity η_w_; the effect of the yield stress is accounted for by a frictional block that slides only if tension is higher than the threshold for sliding. The characteristic time of the wall relaxation is the ratio of viscosity to stiffness, τ_w_ = η_w_/K_w_; more precisely, under constant strain, the stress in the wall relaxes exponentially to 0 with a time-constant τ_w_. We hereafter use this model to compute the response of the cell wall to external loading.

#### Two Categories of Sensors

The sensors respond to mechanical signals through their deformations and we model them as springs. In the absence of detailed knowledge on the modes and kinetics of association of sensors to the cell wall, it would be difficult to systematically consider the role of all possible combinations of modes and kinetics in sensing the mechanical status of the wall. Nevertheless, we could make progress by making the reasonable assumption that the sensor has negligible direct impact on the cell wall mechanical properties. In other words, we assumed the force in the sensor to be small compared to the stress in the cell wall. Under this assumption, we may consider the sensor as an addition to the cell wall and model it in a mechanical branch parallel to the cell wall (Figure 4A, 5A). Two main cases arise.

In the first case, the sensor is associated with a minor load-bearing structure, either a cell wall component or the plasma membrane. We consider the sensor as a spring in series with this structure, the spring and the structure being parallel to the major portion of cell wall because they carry a small fraction of stress (Figure 4A). Stress is released in the structure with which the sensor is associated to, due to cell wall remodeling or to membrane turnover. In such a case, the mechanoperception of the sensor may depend on the kinetics of its association. We make the reasonable assumption that the sensor binds quickly to the structure, so that structure relaxation (remodeling/turnover) is slow compared to binding, while the lifetime of the sensor is much longer than its binding time. Releasing this assumption would not affect our conclusions, but only the magnitude of sensing.

In the second case, the sensor is associated with a major load-bearing component of the cell wall, like cellulose, or possibly hemicellulose segments that cross-link cellulose fibers. We consider the sensor as a spring parallel to a segment of the whole cell wall (Figure 5A). In this case, stress in the sensor continuously increases as the medium expands and the sensor detaches or dissociates when the force applied to the sensor reaches a threshold.

### Response of a Sensor Associated With a Minor Load-Bearing Structure

#### Model

We now consider a sensor that is associated to a minor load-bearing structure such as the plasma membrane or a subpart of the cell wall (Figure 4B). We model the sensor as a spring and the structure as a viscoelastic material. The combined elastic properties of the structure and of the sensor are described by a stiffness K_s_, while the viscous behavior of the structure is described by a viscosity η_s_, corresponding to a branch with a spring and a dashpot in series, the whole being in parallel with the remainder of the cell wall, as shown in Figure 4A. A timescale τ_s_ = η_s_/K_s_ characterizes the transition from elastic to viscous behavior of the branch representing the sensor-structure continuum; τ_s_ corresponds to the time required for this branch of the cell wall, including the sensor, to remodel and relax its stress. We found that the ratio of this time scale, τ_s_, to that of the cell wall, τ_w_, is an important parameter for sensing. For instance, if τ_s_ is much smaller than τ_w_, then the stress in the sensor-structure branch relaxes more quickly than in the remainder of the cell wall. Note that the cell wall relaxation results from the relaxation of all its constituents and so the relaxation time of the wall is always greater than that of any of its constituents, and therefore τ_s_ cannot be greater than τ_w_.

Hereafter, we present our results concerning the response of the sensor to stress and to strain (details of calculations in Supplementary Material). In each case we consider a mechanical signal (or input) as a function of time measured relatively to the relaxation time of the sensor-structure continuum (*t*/τ_s_). This input, shown as a blue line, is either stress (Figure 4C,D) or strain (Figure 4E). The input has a constant background, which corresponds to turgor-generated stress in the wall or to the expansion rate of the wall, and a transient peak, which corresponds to a transient external force like the transient bending of a stem. The output of the model is the force transmitted to the sensor; it is represented as a function of time and is shown as a green line (Figure 4C–E). This output represents the “amount of information” from the input that is “accessible” to the sensor and downstream signaling.

#### Stress Response

We consider a growing cell wall for which the wall tension is higher than Y (Figure 4C,D). The information accessible to the sensor depends on the magnitude of the local relaxation time compared to the wall relaxation time τ_s_. We first examined the case where τ_s_ and τ_w_ have the same magnitude (τ_s_ ≃ τ_w_). We found that the force in the sensor is proportional to the wall stress: as shown in Figure 4C, the output overlaps the input. Accordingly, both constant and transient components of the stress signal are accessible to the sensor, which can thus sense both fast and slow variations of the stress.

We then examined the case where τ_s_ is much smaller than τ_w_ (τ_s_⟨⟨τ_w_), which can be illustrated by a sensor inserted in the plasma membrane. We found that slow variations of the stress (constant component of the signal) cannot be perceived by the sensor since, in the branch where the sensor is, the stress has time to relax. This is illustrated in Figure 4D by the very low value of output away from the peak time. In such a case, the sensor is not sensitive to slow variations of wall stress, but is still sensitive to stress variations that are faster than τ_s_, as shown in Figure 4D by a transient output peak of the same amplitude as the input peak.

We next considered a non-growing cell wall for which the wall tension is below the yield stress Y. In this regime, wall viscosity and relaxation time are infinite. Slow variation of the stress cannot be perceived because of local relaxation. The sensor is then only sensitive to transient variations of the stress that are faster than τ_s_ as in the preceding case (see Supplementary Material).

Finally, we examined robustness of sensing to fluctuations (due to spatial or temporal heterogeneity) of cell wall mechanics. We found that the response of a sensor only depends on ratios of local mechanical properties (modulus and/or viscosity) to cell wall scale properties and that the sensor may also respond to changes in these ratios when the cell wall is under stress (see Supplementary Material).

Altogether, we propose that a sensor associated with a minor load-bearing structure is always sensitive to variations of stress, and is also sensitive to permanent stress when the wall is growing and local relaxation rate is comparable to wall relaxation. However, such a sensor may also respond to changes in cell wall structure.

#### Growth/Strain Response

We then considered the response of the sensor to strain. This differs from the response to stress in the sense that in the case of the response to strain, the deformation is imposed to the system, while in the case of the response to stress, a stress is applied and the deformation in the system depends on the viscoelastoplastic properties of the modeled cell wall. Thus in this case τ_w_ becomes irrelevant because the two branches of the model are deformed at the same rate. Whereas the deformation is thus imposed in the branch of the sensor, it remains that the elastic deformation in this branch is released over time by the viscous component of the branch (which is due to the structure to which the sensor is associated). The rate of this release is the characteristic time τ_s_ of the sensor-structure continuum and thus the sensor is insensitive to variations of strain rate faster than τ_s_. Accordingly, we found that the force in the sensor is proportional to the wall expansion rate averaged over τ_s_. As can be seen in Figure 4E, the output is significant in the constant input regime, whereas the input peak is smoothed due to averaging over a characteristic time τ_s_.

We examined robustness of sensing to fluctuations of cell wall mechanics. The response of one sensor actually only depends on local viscosity and therefore would be sensitive to fluctuations in plasma membrane and/or matrix viscosity, yielding a slightly more robust response to strain than to stress (see previous subsection).

Altogether, we propose that a sensor associated to a minor load-bearing structure responds to permanent growth rate and averages changes in growth or transient external strain over a timescale associated with structure turnover or remodeling. However, such a sensor may also respond to changes in structure viscosity.

### Response of a Sensor Associated With a Major Load-Bearing Structure

#### Model

We now consider a sensor that is associated to a major load-bearing structure such as cellulose microfibrils (Figure 5B). This model differs from the previous one in that in this case, the “branch” of the sensor is solely composed of the sensor, while in the previous case the sensor was considered as inserted in a sub-fraction of the wall with potentially rheological properties that differ from those of the whole cell wall. Accordingly, the sensor follows the stretching of the whole cell wall. Since we consider that the sensor is not infinitely stretchable, we assume that after reaching a certain extension, the sensor undergoes dissociation and is no more exposed to the mechanical signal of the cell wall, until it possibly re-associates with the cell wall in an un-stretched state. We model such a sensor as a spring of strength k_s_ and length l parallel to a segment of the cell wall, the whole being in series with the remainder of the cell wall. This segment is assumed to be initially at rest, and has elastic and viscous coefficients of this segment k_w_ and α_w_ as shown in Figure 5A. Given that this segment has a length l, the elastic and viscous coefficients can be approximated as k_w_ ≃ K_w_/l, α_w_ ≃ η_w_/l. As stated above, we assume the sensor much softer than the wall (k_s_⟨⟨k_w_).

The sensor increases in length while it is intact and bound to the cell wall. We model its dissociation rate, 1/τ_d_, as an increasing function of the force in the sensor, *F*: 1/τ_d_ = 1/τ_c_f(F/F_c_), where τ_c_ is the maximal life time of the sensor that corresponds to F_c_ the typical force above which the sensor dissociates. The behavior of the sensor will depend on the rate at which it is loaded with respect to 1/τ_c_. For fast loading, the sensor dissociates at the maximal force F_c_ and its lifetime is determined by the time needed to reach this maximal force, a time that is determined by the stress in the wall; accordingly, mechanosensing requires downstream signaling to be sensitive to the rate at which sensors detach. In contrast, for slow loading, the force in the sensor remains small with respect to F_c_ and the rate of detachment is approximately constant and equal to 1/τ_c_; at detachment, the force in the sensor is related to wall stress; accordingly, mechanosensing requires downstream signaling to be sensitive to the force in the sensor when it detaches. Hereafter, we present the results for the response to stress and to strain (details of calculations in Supplementary Material), making the distinction between fast and slow loading. As for the first category of sensors, we consider a mechanical signal (input, blue line) with a constant component and a transient increase (Figure 5C–H). The output (green line) is, according to cases, either the actual force (normalized by F_c_) at which the sensor dissociates or the rate of dissociation (normalized by 1/τ_c_).

#### Stress Sensing

First, we investigate the response of the sensor to stress, which may be the permanent stress associated with turgor or variations in stress due to turgor changes or to external stress application. The mechanoperception of this type of sensor depends on the magnitude of the dissociation force F_c_ (the typical force above which the sensor dissociates) relative to the wall tensionσ and its variations. If F_c_ is large compared to (k_s_/k_w_)σ, which means that the sensor remains attached even under high force, then the dissociation of the sensor occurs at a rate 1/τ_c_, which is the usual turnover rate of the sensor as defined above. In this case, the force applied to the sensor depends on the level of stress in the cell wall. Two subcases arise according to the maximal lifetime of the sensor.

If the maximal lifetime, τ_c_, is comparable to or greater than the relaxation time, τ_w_, then the cell wall may significantly relax while the sensor is active, and the cell wall shows both elastic and viscous behaviors. For this reason, we find that if the wall stress has both slow and fast variations (blue line in Figure 5C), then the sensor stretches under the influence of the permanent load. On top of this permanent stretching, the sensors detaching as the wall is transiently pulled are more stretched because of the elastic behavior of the cell wall, which gives rise to a first peak, whereas those sensors which are deposited as the wall is transiently pulled will later detach with a lower stretching. Sensors detaching as the wall is transiently pulled have an increased stretching, which explains the first peak of the green line in Figure 5C and sensors deposited as the wall is transiently pulled, later detach with a lower force, explaining the transient decay of the sensor force represented in Figure 5C. Altogether, long-lived sensors with high dissociation force respond to permanent stress and to variations of stress, and the response to a stress peak shows a peak followed by a dip.

If the maximal lifetime, τ_c_, is small compared to the relaxation time of the cell wall, τ_w_, then the cell wall does not significantly relax while the sensor is active and the cell wall appears elastic over this time window. Accordingly, the sensor is only sensitive to transient variations of the wall stress that are faster than τ_c_. If the wall stress has both slow and fast variations (blue line in Figure 5D), the level of force at which the sensor detaches only follows fast variations as represented by the green curve in Figure 5D which shows the force in the sensor as it detaches as function of the time they detach. Sensors which detach as the wall is transiently pulled will therefore be stretched and have higher force. Also, sensors which are deposited as the wall is transiently under tension will later be stretched which explains the second peak of the output (Figure 5D). Sensors which are deposited before the wall is transiently pulled and which detach after are temporarily stretched but detach in an unstretched state. Altogether, short-lived sensors with high dissociation force respond only to variations of stress, and the response shows two consecutive peaks.

We now move to the case where dissociation force, F_c_, is low compared to (k_s_/k_w_)σ, whereby the sensor detaches as its force reaches F_c_ and the dissociation rate of the sensor depends on the stress in the wall. Accordingly, mechanosensing would require the downstream signal to be sensitive to the rate of sensor turnover and we consider as an output the dissociation rate. Sensing then depends on both viscous and elastic behavior of the cell wall.

If the wall is in an elastic regime (for instance, below the yield stress), the sensor will be stretched by variations of the wall stress occurring in a time shorter than τ_c_. The greater the variation of the stress, the faster the sensor detaches. Our results are represented in Figure 5G where the green line shows the normalized dissociation rate of a sensor as function of the time at which it detaches, for an input wall stress that follows the blue curve. The frequency of dissociation is maximal when the wall stress variation is maximal. If the wall stress variation is too low, the dissociation rate equals the minimal value 1/τ_c_. Dissociation rate is maximal when the variation of wall stress is maximal; the sensor then responds to both increase and decrease of stress, which explains the two peaks in the output (Figure 5G); the discontinuity in the output can be ascribed to the shift from increasing to decreasing stress. Altogether, sensors with low dissociation force respond to variations of stress of a non-growing wall, and the response to a stress peak shows two peaks.

When the cell wall behaves as a viscous material (for instance, above the yield stress), the stretching of the sensor increases under the effect of the irreversible expansion of the cell wall. The rate of dissociation of the sensor 1/τ_d_ will then depend on the wall stress averaged over a time τ_d_. As shown in Figure 5F. where the green line represents the dissociation rate 1/τ_d_ as function of the time when the wall stress follows the time evolution represented by a blue curve. Altogether, sensors with low dissociation force show a smoothed (integrated) response to variations of stress of a growing wall, and the response to a stress peak shows a single peak.

Finally, we examined the robustness of mechanosensing to fluctuations in the mechanical properties of the cell wall. We found that, like for the other category of sensors, the sensor may respond to variations in mechanical properties.

#### Growth/Strain Sensing

Second, we investigate the response of the sensor to steady growth, or to variations associated with changes in growth rate or with externally applied deformation. In any case, the extension of the sensor follows the extension of the wall because the sensor is much softer than the wall. The typical force in the sensor is therefore proportional to the cell wall rate of elongation (be it reversible or irreversible) and to the dissociation time τ_d_. If the dissociation force τ_c_ is high, then the dissociation time approximately equals τ_c_, and the sensor will tend to smooth wall strain rate over a time τ_c_. This is illustrated in Figure 5E, which shows in green the force of dissociating sensors as function of the time at which they dissociate, for an imposed wall expansion rate represented in blue. If the dissociation force F_c_ is low, then dissociation time will depend on the averaged level of stress. If the wall stress is high enough, the sensor will dissociate rapidly and the smoothing will be on a shorter time as illustrated in Figure 5H. It shows by a green line the normalized dissociation rate of sensors as a function of the time at which they dissociate.

Altogether, the sensor responds to strain rate, with more integration (smoothing) when the dissociation force is high. In contrast with previous cases, the response of the sensor is insensitive to cell wall mechanical properties and so is very robust.

## Discussion

Here, we presented the concepts associated with mechanosensing of the plant cell wall, we briefly reviewed putative mechanosensors, and we built simplified biophysical models of sensing. We found that, in these models, the response depends on how the mechanosensor is associated with the cell wall. We explained how a sensor could be sensitive to transient variations of the wall stress or the smooth variations depending on the relaxation of the medium in which it is inserted or on the kinetics of dissociation of the sensor. We can summarize our findings as follows. Sensors are in general sensitive to transient stresses, but, to sense permanent stresses, their dynamics must be slow enough. This applies to sensors associated with minor load-bearing structures that relax slowly, such as sensors inserted into the pectin matrix. In contrast, sensors inserted in the plasma membrane, such as the stretch-sensitive ion channels should only be sensitive to transient variations of the stress. Sensors associated with major load-bearing structures sense permanent stress if their dissociation kinetics is slow enough. This may correspond to relatively stiff and tightly cross-linked components of the cell wall (polysaccharides or proteins cross linked to the cell wall such as the HRGP). On the other hand, sensors that have a small dissociation force can be sensitive to both permanent and transient stress in the context of a growing wall, but are insensitive to permanent stress and very sensitive to variations of the stress in the context of an elastic wall (non-growing). This case may correspond for example to peptides weakly bound to the cell wall.

Concerning the response to strain the picture is more simple. We could show that growth sensing is smoothed for the two types of sensor that we described. Thus sensors are always sensitive to constant growth rate but are relatively less sensitive to growth rate variations.

Finally we could show that in almost all cases, the sensors are sensitive to fluctuations in the cell wall mechanics (e.g., due to active cell wall synthesis, secretion and remodeling), potentially affecting their stress and strain sensing capacities.

So, do plants sense stress or strain? In fact our minimal models do not really allow us to directly answer this question. First because we only tested a few cases of minimal theoretical sensors, but mostly because our approach is not meant to inform us on whether stress and/or strain are actually sensed but on whether this information is accessible to the sensor. In other words, we can only conclude on what cannot the sensed by the sensor, provided the model used is realistic enough. Our model addresses neither downstream transduction of the signal into morphological consequences, nor its effects on, e.g., cytoskeleton or ion influx. Nevertheless, our model can inform us on how much signal and which type of information can be, in principle, transmitted downstream. Such signal could be further filtered or buffered by downstream signaling modules and cellular components, contributing to robust morphogenesis. Nevertheless, we believe that our approach will provide a useful framework to investigate potential mechanosensing mechanisms in plants and whether they could in principle allow the sensing of the stress and\or the strain as well as their constant and transient components.

## Data Availability

No datasets were generated or analyzed for this study.

## Author Contributions

AB initiated to this work. SV reviewed the biological knowledge. AF, SV, and AB designed the models. AF implemented and analyzed the models. AF, SV, and AB wrote the manuscript.

## Conflict of Interest Statement

The authors declare that the research was conducted in the absence of any commercial or financial relationships that could be construed as a potential conflict of interest.
