# Supplementary material for: Feeling Stressed or Strained? A Biophysical Model for Cell Wall Mechanosensing in Plants
Source: Front Plant Sci. 2019 Jun 12;10:757. doi: 10.3389/fpls.2019.00757 (PMC6581727; doi:10.3389/fpls.2019.00757)
Supplement: Supplementary file 1 [file Data_Sheet_1.PDF]

Supplementary Material: Feeling stressed or strained? A biophysical model for cell wall mechanosensing in plants

Antoine Fruleux, Stéphane Verger, and Arezki Boudaoud

We present one-dimensional models for sensors associated with a major or a minor load-bearing structure, and analyse their response to stress and strain rate.

1 CELL WALL

We model the cell wall as a viscoelastoplastic material, represented by a spring of elastic modulus K_w in series with a dashpot of viscosity η_w in parallel with a frictional block of yield stress Y (Fig. 3). The cell wall dynamics is governed by

$$\frac{\dot{\sigma}}{K_\mathrm{w}} + \frac{\sigma(t) - Y}{\eta_\mathrm{w}} = \gamma, \tag{S1}$$

where σ is the cell wall tension, the dot stands for the derivative with respect to time t, and γ is the strain rate that combines growth (irreversible expansion) and elastic deformations. The comparison of the viscous and the elastic term in (S1) yields a relaxation time $\tau_\mathrm{w} = \eta_\mathrm{w}/K_\mathrm{w}$. Y is the yield stress above which the wall starts to show viscous properties. η_w therefore depends on the wall tension σ and the threshold Y,

$$\eta_\mathrm{w} = \left| \begin{array}{lll} \eta_0 & \text{if} & \sigma > Y, \\ +\infty & \text{if} & \sigma < Y. \end{array} \right. \tag{S2}$$

For simplicity, we also consider the stress in excess of the theeshold $\tilde{\sigma} = \sigma - Y$.

2 SENSOR ASSOCIATED WITH A MINOR LOAD-BEARING STRUCTURE

2.1 Model

The sensor is assumed to be inserted into a visco-elastic medium in parallel to the wall (Figs. 4A,B). An elastic modulus K_s accounts for the effective elastic properties of the sensor and the structure in which it is inserted, whereas a viscous coefficient η_s accounts for the viscosity of the structure. The relaxation time above which the medium exhibit viscous behaviour is $\tau_s = \eta_s/K_s$. Given that the cell wall has a relaxation dominated by its slowest component, it is expected that τ_s is at most comparable in magnitude to τ_w.

The impedance of the structure in which the sensor is assumed small. Therefore, the force exerted on the sensor F is small as compared to the force in the overall cell wall σ, and is governed by

$$\frac{\dot{F}(t)}{K_s} + \frac{F(t)}{\eta_s} = \frac{\dot{\sigma}(t)}{K_\mathrm{w}} + \frac{\tilde{\sigma}(t)}{\eta_\mathrm{w}}. \tag{S3}$$

2.2 Response to a stress

We first study the response of such sensor to stress. Equation (S3) yields

$$F(t) = \frac{\eta_s}{\eta_w}\left[\frac{\tau_w}{\tau_s}\Delta_{\tau_s}\tilde{\sigma}(t) + \langle\tilde{\sigma}(t)\rangle_{\tau_s}\right] \tag{S4}$$

where

$$\langle\tilde{\sigma}(t)\rangle_{\tau_s} = \int_{-\infty}^{0} \frac{d\tau}{\tau_s}\, e^{\frac{\tau}{\tau_s}}\, \tilde{\sigma}(t+\tau) \tag{S5}$$

is the slow tend of stress $\tilde{\sigma}$ and

$$\Delta_{\tau_s}\tilde{\sigma}(t) = \tilde{\sigma}(t) - \langle\tilde{\sigma}(t)\rangle_{\tau_s} \tag{S6}$$

contains its fast variations. If the structure relaxes much faster than the cell wall ($\tau_s \ll \tau_w$), slow fluctuations are filtered out. Assuming stress fluctuations comparable in magnitude to average stress, the sensor is then mainly sensitive to the fast fluctuations of the stress (of timescales under τ_s). If the structure and the wall have similar relaxation times ($\tau_s \sim \tau_w$), then the sensor is sensitive to both fast and slow wall stress fluctuations.

2.3 Response to growth

We now study the response of such sensor to strain rate. The relation between the force exerted on the sensor and the expansion rate γ is found to be

$$F(t) = \eta_s\langle\gamma(t)\rangle_{\tau_s} \tag{S7}$$

where $\langle\gamma(t)\rangle_{\tau_s}$ is the expansion rate where fast variations (with timescales below τ_s) are filtered, defined as in Eq. (S5).

3 SENSOR ASSOCIATED WITH A MAJOR LOAD-BEARING STRUCTURE

3.1 Model

3.1.1 Constitutive law

We model this wall sensor as a spring of strength k_s in parallel with a segment of the cell wall. We write the elastic and viscous coefficients of the segment of the wall k_w and α_w. If l_s is the size of the wall sensor, then we estimate $k_\mathrm{w} \sim \frac{K_\mathrm{w}}{l_s}$, $\alpha_\mathrm{w} \sim \frac{\eta_\mathrm{w}}{l_s}$, and $\frac{\alpha_\mathrm{w}}{k_\mathrm{w}} \sim \tau_\mathrm{w}$. For simplicity, we consider $\alpha_\mathrm{w}/k_\mathrm{w} = \tau_\mathrm{w}$ in the following. We make the reasonable assumption that the sensor is much less stiff than the wall, so that $F \ll \sigma$. As a result, wall expansion forces the extension of the sensor, so that the the force in the sensor is governed by

$$\frac{\dot{F}}{k_s} = \frac{\dot{\tilde{\sigma}}}{k_\mathrm{w}} + \frac{\tilde{\sigma}}{\alpha_\mathrm{w}}. \tag{S8}$$

3.1.2 Dissociation

The sensor is stretched by the cell wall until it dissociates. The dissociation rate $1/\tau_d$ of the sensor must depend on the force in the sensor $1/\tau_d = 1/\tau_c f(F/F_c)$, where τ_c is the characteristic dissociation rate and F_c is the typical force above which dissociation systematically occurs. If the sensor is able to reach forces

of magnitude $\sim F_c$ in times smaller than τ_c, then wall tension is mostly reflected by the lifetime τ_d of the sensor, else the wall tension is mostly reflected by the force at which the sensor dissociates.

3.2 Response to stress

We first study the response of such sensor to stress. The relation between the force F exerted on a sensor associated at time t_a and the stress of the cell wall is deduced from Eq. (S8):

$$F(t) = \frac{k_s}{k_\mathrm{w}} \left[\{\tilde{\sigma}(t) - \tilde{\sigma}(t_a)\} + \frac{t - t_a}{\tau_\mathrm{w}} \langle\langle\tilde{\sigma}(t)\rangle\rangle_{t-t_a} \right], \tag{S9}$$

where

$$\langle\langle\tilde{\sigma}(t)\rangle\rangle_{t-t_a} = \int_{t_a-t}^{0} \frac{d\tau}{t - t_a} \tilde{\sigma}(t + \tau) \tag{S10}$$

is the stress smoothed with a resolution $t - t_a$. The sensor response to mechanical signals depends on the value of the dissociation force as discussed in the next subsections.

3.2.1 High dissociation force

Let $\delta_{\tau_c}\tilde{\sigma}$ be the magnitude of wall stress fluctuations at a timescale smaller than τ_c. If $F_c \gg k_s/k_\mathrm{w}\delta_{\tau_c}\tilde{\sigma}$ and $F_c \gg k_s/k_\mathrm{w}\,\tau_c/\tau_\mathrm{w}\langle\langle\tilde{\sigma}(t)\rangle\rangle_{t-t_a}$, then the sensor detaches after $\tau_d \sim \tau_c$, with a force

$$F(t) \sim \frac{k_s}{k_\mathrm{w}} \left[\{\tilde{\sigma}(t) - \tilde{\sigma}(t - \tau_c)\} + \frac{\tau_c}{\tau_\mathrm{w}} \langle\langle\tilde{\sigma}(t)\rangle\rangle_{\tau_c} \right]. \tag{S11}$$

For $\tau_c/\tau_\mathrm{w} \ll 1$, the sensor is sensitive to fast variations of the wall stress, which correspond to the elastic behaviour of the cell wall. In contrast, for $\tau_c/\tau_\mathrm{w} \gg 1$, the sensor is sensitive to slow variations of the wall stress.

3.2.2 Low dissociation force

For low dissociation force, when $F_c \ll k_s/k_\mathrm{w}\,\delta_{\tau_c}\tilde{\sigma}$ or $F_c \ll k_s/k_\mathrm{w} t_c/\tau_\mathrm{w}\langle\langle\tilde{\sigma}(t)\rangle\rangle_{\tau_d}$, then the sensor dissociates when $F \sim F_c$. In this case, the sensor is very sensitive to fast variations of wall stress. When the wall stress $\delta_{\tau_d}\tilde{\sigma}$ varies much faster than τ_w, the dissociation frequency is $1/\tau_d \sim 1/\tau_\sigma\, k_s/k_\mathrm{w}\,\delta_{\tau_d}\tilde{\sigma}/F_c$. When the wall stress varies much slower than τ_w, the rate of dissociation is sensitive to such slow variations, $1/\tau_d \sim 1/\tau_\mathrm{w}\, k_s/k_\mathrm{w}\,\langle\langle\tilde{\sigma}(t)\rangle\rangle_{\tau_d}/F_c$.

3.3 Response to growth

We now study the response to strain rate.The relation between the force exerted on a sensor F associated at a time t_a and the growth rate of the cell wall is deduced from Eqs. (S1,S9):

$$F(t) = (t - t_a)k_s\langle\langle\gamma(t)\rangle\rangle_{t-t_a}, \tag{S12}$$

where $\langle\langle\gamma(t)\rangle\rangle_{t-t_a}$ is the expansion rate smoothed at timescale $t - t_a$, like in Eq. (S10). Depending on the value of the dissociation force, the sensor will respond differently to mechanical signals. For high dissociation force $F_c \gg \tau_c k_s\langle\langle\gamma(t)\rangle\rangle_{\tau_c}$, the sensor dissociates for a force $F(t) \sim \tau_c k_s\langle\langle\gamma(t)\rangle\rangle_{\tau_c}$, after a time $\tau_d \sim \tau_c$. For low dissociation force, $F_c \ll \tau_c k_s\langle\langle\gamma(t)\rangle\rangle_{\tau_c}$, the sensor dissociates for a force $F \sim F_c$ and the frequency of dissociations is $1/\tau_d \sim k_s\langle\langle\gamma(t)\rangle\rangle_{\tau_d}/F_c$.
